# The dissolution, reassembly and further clearance of amyloid‐β fibrils by tailor‐designed dissociable nanosystem for Alzheimer's disease therapy

**DOI:** 10.1002/EXP.20230048

**Published:** 2023-11-23

**Authors:** Qianhua Feng, Xueli Zhang, Nan Zhang, Huan Gu, Ning Wang, Jing Chen, Xiaomin Yuan, Lei Wang

**Affiliations:** ^1^ School of Pharmaceutical Sciences Zhengzhou University Zhengzhou China; ^2^ Henan Key Laboratory of Targeting Therapy and Diagnosis for Critical Diseases Zhengzhou China; ^3^ Department of Chemistry, Chemical and Biomedical Engineering University of New Haven West Haven USA

**Keywords:** Alzheimer's disease, amyloid‐β, dissociable nanosystem, neuroinflammation

## Abstract

The fibrillation of amyloid‐β (Aβ) is the critical causal factor in Alzheimer's disease (AD), the dissolution and clearance of which are promising for AD therapy. Although many Aβ inhibitors are developed, their low Aβ‐binding affinity results in unsatisfactory effect. To solve this challenge, the Aβ sequence‐matching strategy is proposed to tail‐design dissociable nanosystem (B6‐PNi NPs). Herein, B6‐PNi NPs aim to improve Aβ‐binding affinity for effective dissolution of amyloid fibrils, as well as to interfere with the in vivo fate of amyloid for Aβ clearance. Results show that B6‐PNi NPs decompose into small nanostructures and expose Aβ‐binding sites in response to AD microenvironment, and then capture Aβ via multiple interactions, including covalent linkage formed by nucleophilic substitution reaction. Such high Aβ‐binding affinity disassembles Aβ fibrils into Aβ monomers, and induces the reassembly of Aβ&nanostructure composite, thereby promoting microglial Aβ phogocytosis/clearance via Aβ receptor‐mediated endocytosis. After B6‐PNi NPs treatment, the Aβ burden, neuroinflammation and cognitive impairments are relieved in AD transgenic mice. This work provides the Aβ sequence‐matching strategy for Aβ inhibitor design in AD treatment, showing meaningful insight in biomedicine.

## Graphical abstract

A tailor‐designed dissociable nanosystem can decompose into small nanostructures in brain microenvironment of Alzheimer's disease, and then recognize and bind amyloid‐β for the dissolution, reassembly and further clearance of amyloid‐β fibrils. This nanosystem rescues cognitive and memory impairments in mice for Alzheimer's disease therapy.

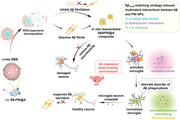

## 1 INTRODUCTION

The toxic β‐sheeted aggregates (e.g. fibrils) of amyloid‐β (Aβ) protein are important steps driving the development of Alzheimer's disease (AD), which induce oxidative stress, neuroinflammation and even neuronal loss.^[^ ^1^ ^]^ With diversified strategies and synergistic therapeutic effects, Aβ‐targeted multifunctional nano‐agents have developed rapidly.^[^ ^2^ ^]^ They can block Aβ aggregation by multiple strategies simultaneously, interfere with multiple pathological factor such as oxidative stress and copper accumulation, integrate various features such as diagnosis and stimuli‐responsive drug release into a single structure. Many Aβ inhibitors are designed to recognize and bind aberrant protein to competitively reduce protein‐protein interactions, thereby depolymerizing Aβ fibrils.^[^ ^3^ ^]^ Among these, nano‐inhibitors with high surface area can provide multiple Aβ binding sites, which have drawn widespread attention.^[^ ^4^ ^]^ Unfortunately, most reported nanostructures bind Aβ via weak noncovalent interaction (hydrophobic interaction, electrostatic binding etc.), making nanostructure only inhibit Aβ monomer fibrillation, however, fail to dissolve the performed amyloid fibrils.^[^ ^5^ ^]^ Collectively, an excellent nanostructure should be designed to satisfy high Aβ‐binding affinity.^[^ ^6^ ^]^ Usually, multivalent binding effect especially covalent attachment can achieve strong binding event. To achieve this goal, nanostructure herein is tailor‐designed with rational chemical design from the perspectives of controllable size, chemical nature, surface chemistry etc.

**SCHEME 1:**
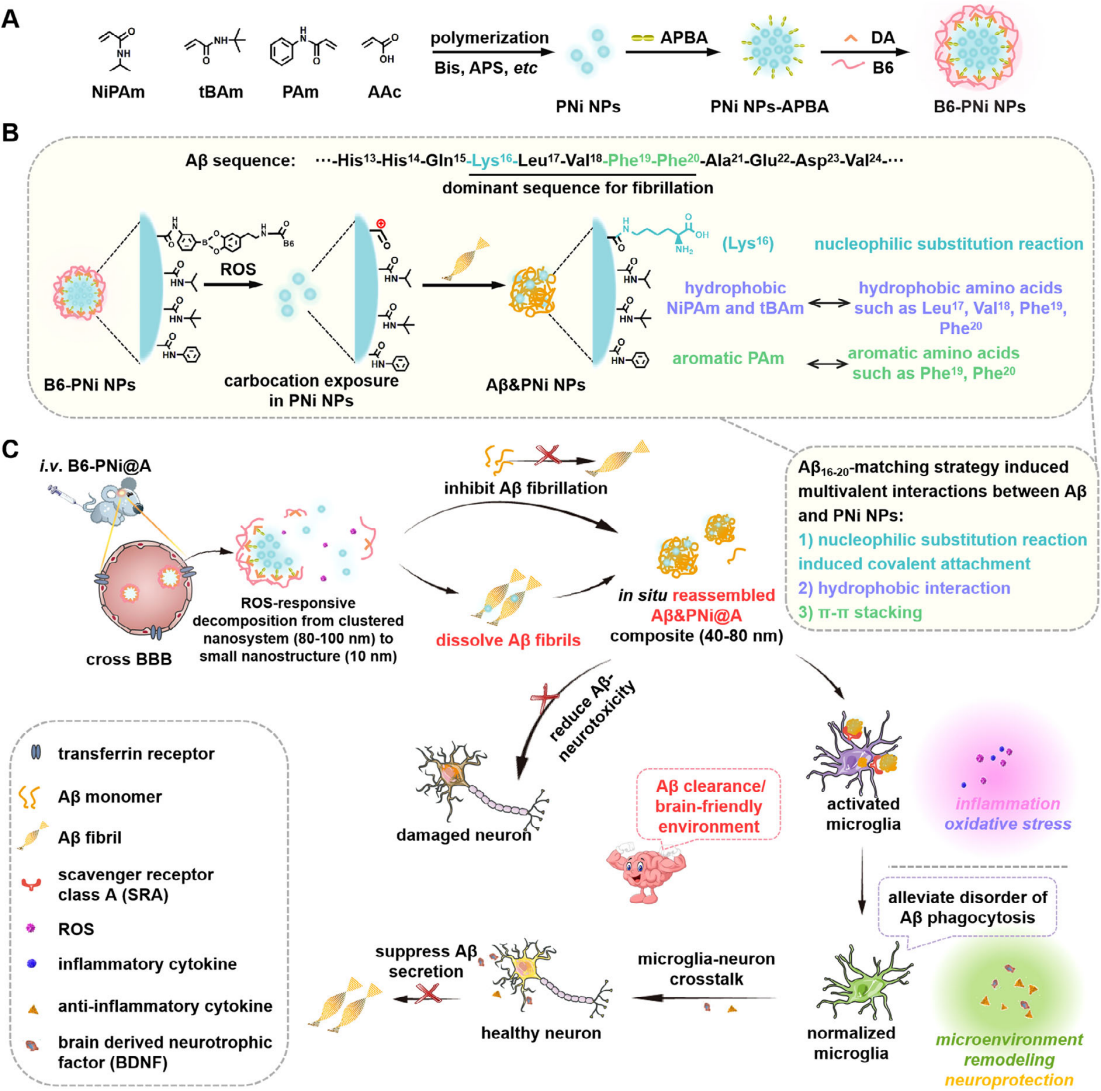
Schematic showing the preparation and therapeutic effect of tailor‐designed dissociable nanosystem. (A) Schematic illustration for the formation of nanosystem (B6‐PNi NPs). (B) The binding mechanism between Aβ and nanostructure (PNi NPs). Based on Aβ_16‐20_‐matching strategy, PNi NPs bind Aβ via multivalent interactions (covalent linkage, hydrophobic interactions, π–π stacking) to form Aβ&PNi NPs. (C) Illustration of the dissolution/reassembly/clearance of Aβ fibrils for AD therapy. B6‐PNi@A crosses BBB via transferrin receptor‐mediated transcytosis. In response to ROS in AD microenvironment, B6‐PNi@A (80–100 nm) decomposes into small PNi@A (10 nm), exposing more binding sites in brain. Then PNi@A dissolves Aβ fibrils via multivalent effect and reassembles into Aβ&PNi@A composite (40–80 nm). Subsequently, Aβ&PNi@A is phagocytized by microglia via SRA. ATV as immunomodulator modulates microglial immunological function to clear Aβ. In addition, the inflammatory AD microenvironment is regulated to a brain‐friendly microenvironment for AD therapy.

Firstly, the 42‐residue Aβ is well‐studied structurally and possesses a dominant sequence (16–20 segments) for fibrillation.^[^ ^7^ ^]^ According to the different properties (hydrophobicity, aromaticity and nucleophilicity etc.) of amino acids in Aβ_16‐20_ (Lys^16^‐Leu^17^‐Val^18^‐Phe^19^‐Phe^20^), it is feasible to design the components of nanostructure to match Aβ_16‐20_ sequence for multivalency effect. Then, in an appropriate proportion, *N*‐isopropylacrylamide (NiPAm) and *N*‐tert‐butylacrylamide (tBAm) with hydrophobicity, *N*‐phenylacrylamide (PAm) with aromaticity, and acrylic acid (AAc) that can produce electrophilic carbocation are integrated into small nanostructures (PNi NPs, 10 nm), which match Aβ_16‐20_ well through hydrophobic interaction and π–π stacking (Scheme 1A,B). Notably, small size of nanostructure can offer more Aβ binding sites, and yet lead to rapid renal clearance.^[^ ^8^ ^]^ Furthermore, controllable covalent interaction in the binding event is being pursued with enthusiasm. To tackle these issues, AD oxidative microenvironment responsive size transformable nanosystem is constructed. The reactive oxygen species (ROS) sensitive 3‐aminophenylboronic acid (APBA) is conjugated onto PNi NPs, which promotes PNi NPs to aggregate into clustered nanosystem (80–100 nm). Then dopamine (DA) and B6 peptide (CGHKAKGPRK) are engineered onto the surface of PNi NPs to obtain B6‐PNi NPs. Such tailor‐designed dissociable nanosystem will improve Aβ binding affinity for effective dissolution of amyloid fibrils due to the following merits: (1) Besides blood‐brain barrier (BBB) crossing ability,^[^ ^9^ ^]^ B6 peptide modification endows PNi NPs with ability to avoid contacting with plasma protein, which increases the possibility of interacting with Aβ in brain. (2) In response to oxidative stress in AD microenvironment, B6‐PNi NPs disintegrate into small nanostructure (PNi NPs, 10 nm) with higher surface area, exposing more Aβ‐binding sites. (3) Based on Aβ_16‐20_‐matching strategy, PNi NPs disassemble Aβ fibrils into Aβ monomer via multivalent interactions (hydrophobic interaction, π–π stacking, covalent attachment). Notably, once APBA structure breaks in response to ROS in AD microenvironment, AAc on PNi NPs can generate electrophilic carbocation to attack nucleophilic lysine^16^ (Lys^16^) in Aβ. Such controllable nucleophilic substitution reaction in vivo generates strong covalent bond between nanostructure and Aβ, which is an innovative design for Aβ fibrillation inhibition (Scheme 1B). More than just dissolving Aβ fibrils, we find that Aβ monomer and nanostructures reassemble into Aβ&nanostructure composite (Aβ&PNi NPs, 40–80 nm) due to strong Aβ‐binding event.

Besides the dissolution of Aβ fibrils, Aβ clearance is also of great significance in AD treatment. However, toxic Aβ fibrils activate microglia to a proinflammatory state, which not only induces Aβ phagocytosis disorder of microglia, but also promotes neuronal Aβ secretion.^[^ ^10^ ^]^ Therefore, modulating microglial function is imperative for Aβ clearance. Herein, atorvastatin (ATV) as immunomodulator is loaded into B6‐PNi NPs to obtain B6‐PNi@A. Interestingly, the in situ reassembly pattern of Aβ&PNi@A in brain achieves microglia‐targeted nano‐delivery via Aβ phagocytic receptor (scavenger receptor class A, SRA)‐mediated endocytosis (Scheme 1C), instead of extra modification of microglia targeting moiety. Subsequently, the reassembled nanocomposite is expected to normalize microglial immunologic dysfunction for Aβ clearance by promoting Aβ phagocytosis, inhibiting neuronal Aβ secretion based on microglia‐neuron crosstalk. Results show that B6‐PNi@A reduces Aβ burden, alleviates microglia activation and rescues memory deficit in mice. Collectively, the tailor‐designed dissociable nanosystem realizes cascaded targeted Aβ and further microglia in brain for dissolution/reassembly/clearance of Aβ fibrils, and additional re‐education of AD microenvironment. This work highlights the key role of size transformation and Aβ sequence‐matching strategy in nanostructure design for high Aβ‐binding affinity.

## 2 RESULTS AND DISCUSSION

### 2.1 Synthesis and size transformation behavior of B6‐PNi NPs

**FIGURE 1:**
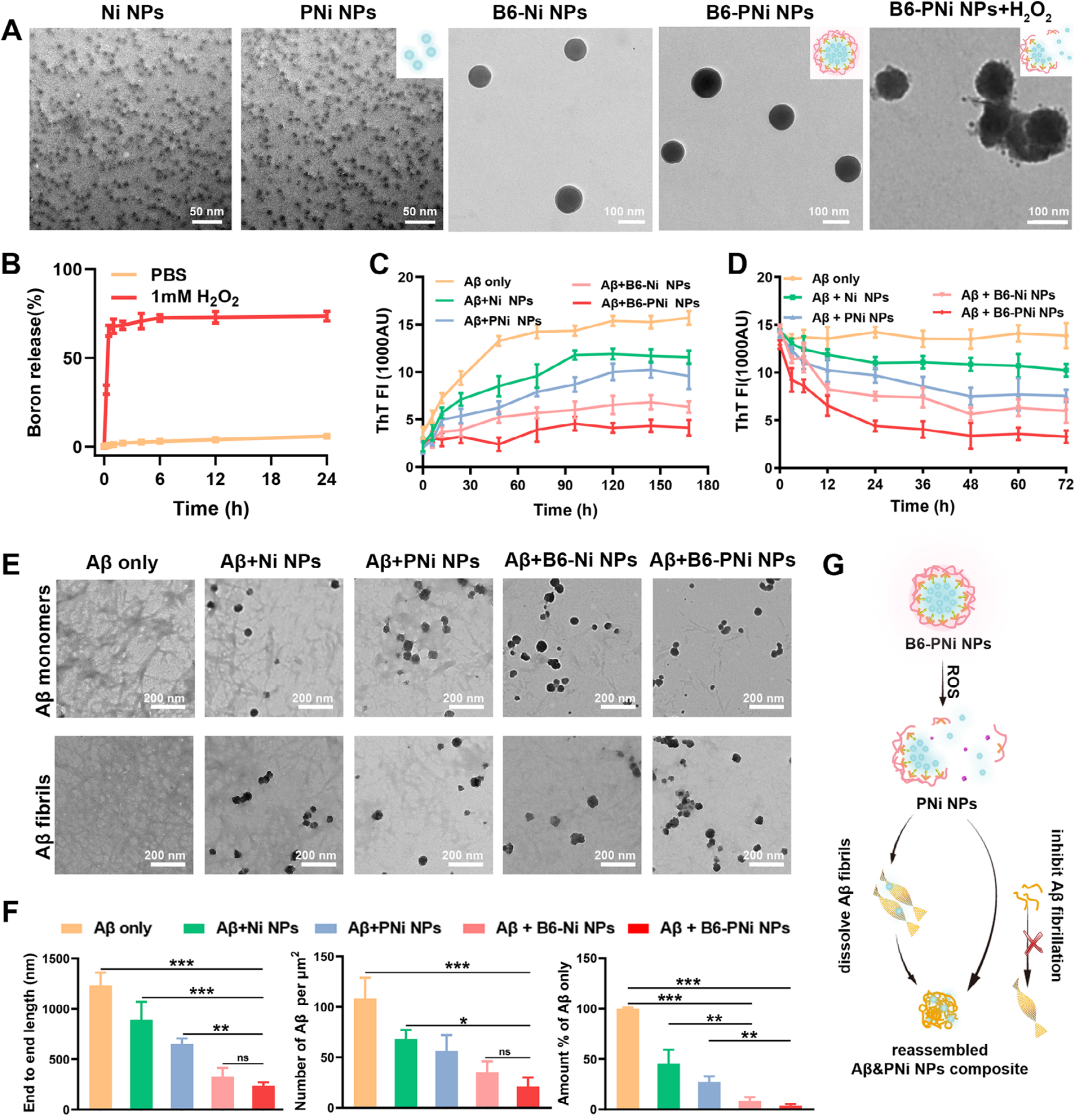
Characterization of B6‐PNi NPs and Aβ fibrillation inhibition effect. (A) TEM images of Ni NPs, PNi NPs, B6‐Ni NPs, B6‐PNi NPs and B6‐PNi NPs in PBS containing H_2_O_2_. (B) Cumulative release profile of boron from B6‐PNi NPs in PBS containing H_2_O_2_ (*n* = 3). (C) Aβ monomers were incubated with nanoparticles to assess Aβ fibrillation kinetics by ThT assay (*n* = 3). (D) Aβ fibrils were incubated with nanoparticles to assess Aβ dissociation kinetics by ThT assay (*n* = 3). (E) TEM images showing the end‐point products of Aβ monomers (top) or fibrils (bottom) in the presence of different nanoparticles (Ni NPs, PNi NPs, B6‐Ni NPs and B6‐PNi NPs), respectively. (F) Quantification of Aβ fibrils per μm^2^ in TEM images (top). (G) Schematic illustration of Aβ&PNi NPs generation. *p < 0.05, **p < 0.01, ***p < 0.001.

Small size of nanoparticle with high surface area can offer more Aβ binding sites. Based on this, NiPAm, tBAm, PAm and AAc with molar ratio of 48:38:2:10 were integrated into small nanostructures (PNi NPs) after a free‐radical polymerization process. The chemical composition of nanostructure was verified by ^1^H NMR (Figure S1), peaks of methyl groups (*δ* = 2.02, 3.85 ppm) and benzene (*δ* = 7.00 ppm) revealed the successful preparation of PNi NPs. Additionally, TEM image showed the uniform morphology with particle size of ≈10 nm (Figure 1A). Then 3‐aminophenylboronic acid (APBA) was conjugated onto PNi NPs via amidation reaction (Figure S2), and its high hydrophobicity led to the aggregation of PNi NPs into clustered nanosystem with increased size (80–100 nm) (Figure S3). Next, dopamine (DA) was grafted onto PNi NPs‐APBA via phenylboronic acid ester bond, and ^1^H NMR (Figure S1) showed that new peaks (*δ* = 7.57, 7.48 ppm) of benzene in DA indicated the reaction. Brain targeted B6 peptide was further engineered onto the surface of PNi NPs, the obtained B6‐PNi NPs dispersed well and the particle size remained unchanged (80–100 nm) (Figure 1A). In addition, to evaluate the role of components of nanoparticle in Aβ‐binding, Ni NPs (10 nm) and B6‐Ni NPs (80–100 nm) were also synthesized without PAm component, respectively. TEM images showed their similar morphology with PNi NPs and B6‐PNi NPs (Figure 1A).

On account of Aβ fibrils mediated inflammatory microglia activation, brain microenvironment of AD accompanies immune dysfunction and exhibits inflammation and high oxidative stress.^[^ ^11^ ^]^ Next, the behavior of B6‐PNi NPs was explored in response to AD microenvironment. B6‐PNi NPs were incubated in PBS containing H_2_O_2_, to simulate pathological oxidative environment in vivo. The amount of released boron in the presence of H_2_O_2_ was quantified by ICP‐MS and reached ≈73.6% after 24 h incubation, far higher than that (5.9%) in the absence of H_2_O_2_ (Figure 1B). These results were assigned to that APBA structure broke in response to ROS.^[^ ^12^ ^]^ Besides, the morphology of B6‐PNi NPs with H_2_O_2_ incubation tended to partially decompose with small particles (10 nm) emerging in the surroundings (Figure 1A), indicating the size transformation from nanosystem (B6‐PNi NPs, 80–100 nm) to small nanostructure (PNi NPs, 10 nm). According to some literature, when the nanoparticles are pre‐coated with an additional protein, the modified protein serves as a protein corona shield, minimizing interactions with serum proteins to prevent the clearance of nanoparticles by macrophages.^[^ ^13^ ^]^ In this work, the B6 peptide modification on PNi NPs is believed to resist interference of plasma proteins to form inevitable “protein corona” during delivery in complex biological environment. Then the AD microenvironment responsive size transformation makes B6‐PNi NPs get rid of B6 peptide protection in brain, thus exposing more binding sites in brain. This precise spatial‐temporal control of binding sites exposure on PNi NPs increases the possibility of Aβ binding.

### 2.2 B6‐PNi NPs inhibited Aβ fibrillation or dissolved Aβ fibrils

To match Aβ sequence, the tailor‐designed PNi NPs were composed of hydrophobic NiPAm and tBAm, aromatic PAm, AAc capable of producing carbocation etc. To assess the roles of components in Aβ binding event, we evaluated four nanoparticles (Ni NPs, PNi NPs, B6‐Ni NPs and B6‐PNi NPs) in Aβ fibrillation process. Herein, Ni NPs and B6‐Ni NPs were employed as comparative nanoparticles without PAm component. To simulate oxidative AD environment in vivo, the following studies were carried out in PBS with the addition of H_2_O_2_. Firstly, thioflavin T (ThT) fluorescence assay was used to investigate fibrillation kinetics of Aβ monomer (Figure 1C).^[^ ^14^ ^]^ In the absence of nanoparticles, a rapid increase in ThT fluorescence intensity (1.57×10^4^) was observed, substantiating Aβ monomer gradually underwent fibrillation. In contrast, Ni NPs, PNi NPs, B6‐Ni NPs and B6‐PNi NPs treatments reduced 26.4%, 39.1%, 59.8% and 73.7% of ThT fluorescence intensity, respectively. Circular dichroism (CD) spectra was used to investigate the fractional secondary structure of Aβ.^[^ ^15^ ^]^ Figure S4 showed B6‐PNi NPs significantly reduced toxic β‐sheet content of Aβ from 50.6% to 13.4%, suggesting its inhibitory effect on Aβ fibrillation. Besides, when nanoparticles were incubated with the performed Aβ fibrils, the decreased ThT fluorescence intensity verified the ability of nanoparticles to dissolve Aβ fibrils (Figure 1D). The differences in Aβ fibrillization inhibition effect among nanoparticles were further assessed by TEM (Figure 1E, top). It showed that Aβ monomers aggregated into fibrils after 7 days of incubation. However, treatments of four nanoparticles induced less and shorter Aβ fibrils in varying degrees. Besides, the length, number and amount of remained Aβ fragments were quantified (Figure 1F). Compared with Aβ only group, B6‐PNi NPs reduced length, number and amount of Aβ fibrils, exhibiting the strongest Aβ fibrillation inhibition effect. Similarly, when incubating with Aβ fibrils for 3 days, four nanoparticles could effectively disassemble Aβ fibrils into fragments (Figure 1E, bottom). The above results indicated that nanoparticles could bind Aβ, thus reducing Aβ‐Aβ interactions to inhibit Aβ fibrillation or dissolve Aβ fibrils.

**FIGURE 2:**
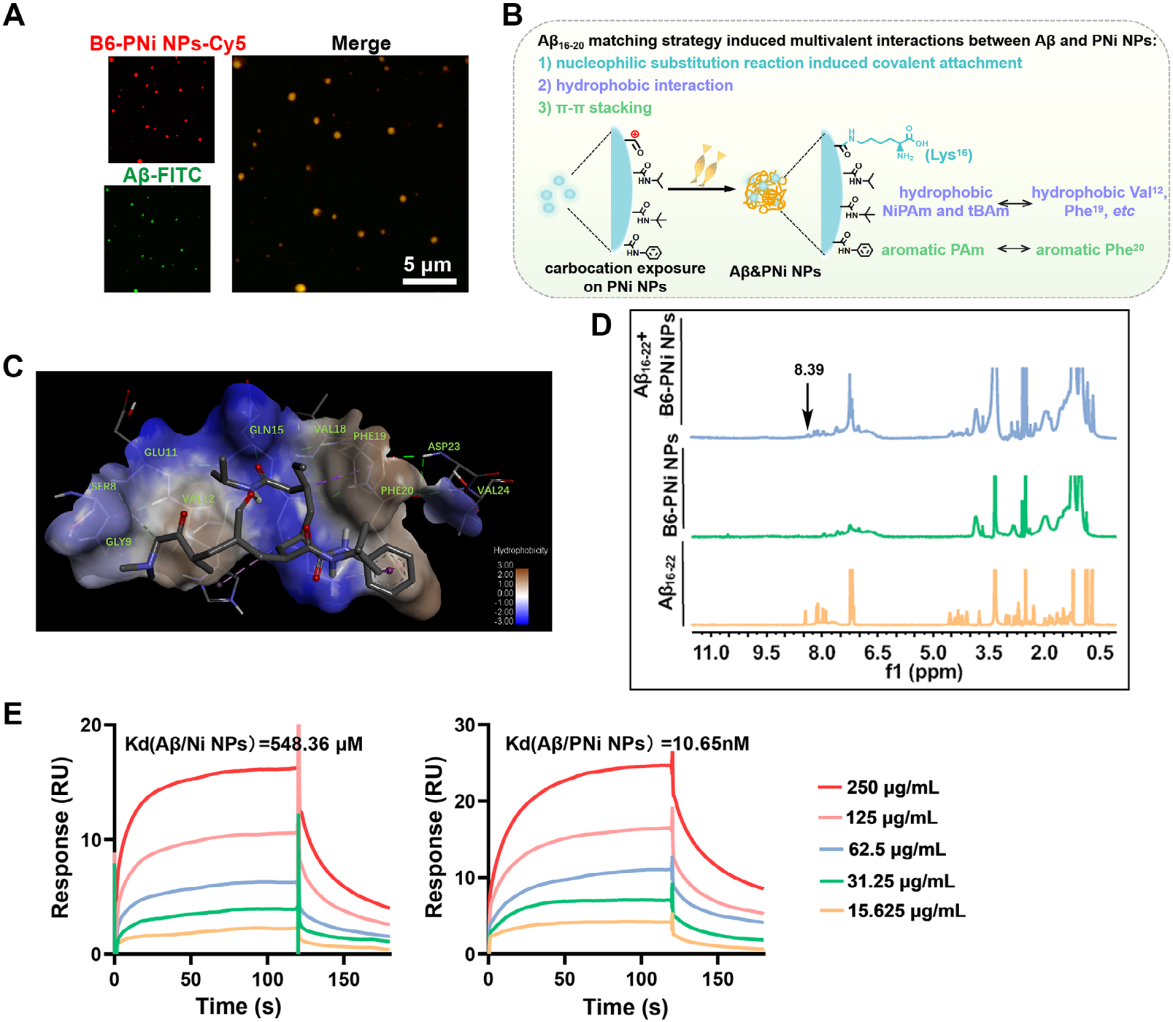
Binding mechanism analysis between Aβ and nanostructure. (A) Fluorescence images showing the end‐point products of FITC labeled Aβ monomers (green) in the presence of Cy5 labeled B6‐PNi NPs (red). (B) Schematic representation of binding mechanism between Aβ and carbocation intermediate contained PNi NPs. (C) For molecular docking simulation, energy‐minimized average model of carbocation intermediate contained PNi NPs‐Aβ interactions. (D) ^1^H NMR spectra of Aβ_16‐22_, B6‐PNi NPs and the product after incubating Aβ_16‐22_ and B6‐PNi NPs in PBS containing H_2_O_2_, respectively. (E) SPR analysis of concentration‐dependent binding of the Ni NPs and PNi NPs to Aβ monomer.

It was noteworthy that spherical nanoparticles (40‐80 nm) were observed in TEM images after treatments of four nanoparticles. Since dissociable nanosystems (B6‐Ni NPs or B6‐PNi NPs, 80–100 nm) decomposed into small nanostructures (Ni NPs or PNi NPs, 10 nm) in PBS containing H_2_O_2_ to expose surface binding sites, we supposed that nanostructures and monomeric Aβ after fibrils dissolution reassembled into Aβ&nanostructure composite (Aβ&Ni NPs or Aβ&PNi NPs, 40–80 nm) (Figure 1G). What is more, the colocalization of Cy5 labeled B6‐PNi NPs (red) and FITC labeled Aβ monomers (green) was visualized, validating Aβ&PNi NPs formation (Figure 2A). Therefore, we confirmed that four nanoparticles especially B6‐PNi NPs inhibited Aβ fibrillation or even dissolved Aβ fibrils, and further reassembled with monomeric Aβ to form Aβ&nanostructure composite.

### 2.3 Binding mechanism analysis between Aβ and nanostructure

The Aβ fibrillization inhibition effects of four nanoparticles (B6‐PNi NPs > B6‐Ni NPs > PNi NPs > Ni NPs) were related to the Aβ‐binding affinity. In this sense, components (NiPAm, tBAm, PAm, AAc etc.) and B6 modification in these nanoparticles might play key roles in the interactions between Aβ and nanoparticles. For B6‐PNi NPs which had highest inhibitory effect in Aβ fibrillation, they decomposed into PNi NPs in response to AD oxidative microenvironment owing to the cleavage of APBA structure. Notably, AAc structure on PNi NPs would convert to electrophilic carbocation. That meant PNi NPs exposed multiple binding sites (AAc derived electrophilic carbocation, aromatic PAm, hydrophobic NiPAm and tBAm etc.) (Scheme 1B, Figure 2B). We then conducted molecular docking simulation between Aβ and the above carbocation intermediate contained PNi NPs (Figure 2C). Results showed hydrophobic components (e.g. NiPAm, tBAm) in PNi NPs mainly captured some hydrophobic amino acids (Val^12^, Phe^19^, Phe^20^ etc.) in Aβ through hydrophobic interactions. The close distance between aromatic PAm group and phenylalanine (Phe^20^) implied their π‐π stacking interactions. Notably, Lys^16^ was close to AAc derived carbocation intermediate (distance was 2.5 Å), which allowed the attack of nucleophilic lysine (Lys^16^) by nucleophilic substitution reaction. The above results helped us understand binding mechanism between B6‐PNi NPs and Aβ. Against dominant aggregation sequence of Aβ (Aβ_16‐20_), the tailor‐designed B6‐PNi NPs captured Aβ to match Aβ_16‐20_, which would result in multivalent binding effect (Scheme 1B, Figure 2B).

The molecular docking simulation results showed the possibility of nucleophilic substitution reaction between B6‐PNi NPs and Lys^16^ in Aβ. To further confirm this, trypsin digestion experiment was carried out to explore the binding site of B6‐PNi NPs on Aβ peptide.^[^ ^16^ ^]^ Trypsin exclusively cleaved C‐terminal to arginine and lysine residues. Matrix‐assisted laser desorption/ionization time of flight mass spectrometry (MALDI‐TOF MS) was used to determine the digestive products (Figure S5). For Aβ alone group, m z^−1^ 637.3, 1337.4, 1326.5, 1085.4 signals corresponded to Aβ_1−5_, Aβ_6−16_, Aβ_17−28_, and Aβ_29−40_, respectively. These results showed that Aβ alone was almost completely degraded after trypsin treatment. In the presence of B6‐PNi NPs, the peak height of Aβ_6−16_ reduced significantly to 20.7% compared to Aβ group. However, there were no significant changes in the height of other peaks (Aβ_1−5_ and Aβ_29−40_). These results indicated that B6‐PNi NPs mainly reacted with Lys^16^ instead of Lys^28^ on Aβ. ^1^H NMR assay was carried out to investigate the structure of product after co‐incubation B6‐PNi NPs with Aβ_16‐20_ in H_2_O_2_ solution. Figure 2D showed that new peak of amide at 8.39 ppm emerged, indicating the potential covalent amide bonds generation between Lys^16^ and AAc. What is more, mass spectrometry was used to determine the reaction. We synthesized AAc‐APBA‐DA as an alternative to B6‐PNi NPs. After reaction, the m z^−1^ 201 signal confirmed the formation of AAc‐Lys (Figure S6). These results were assigned to that AAc‐APBA‐DA structure broke in pathological oxidative environment and generated AAc derived carbocation, which allowed the attack of nucleophilic Lys by nucleophilic substitution reaction. In general, the above results fully proved that B6‐PNi NPs decomposed into PNi NPs, and exposed binding sites to capture Aβ via covalent linkage. The controllable covalent interaction under physiological conditions was expected to improve Aβ‐binding affinity. To investigate the binding ability of B6‐PNi NPs to Aβ, B6‐PNi NPs were incubated with various lysine‐containing proteins and primary amine containing molecules. And protein binding ratio was detected by ELISA assay. Figure S7 showed B6‐PNi NPs possessed the most strongest binding effect with Aβ, and would not interfere with normal proteins. We proposed that B6‐PNi NPs could bind Aβ_16‐20_ via hydrophobic interaction and π–π stacking firstly, and then the short distance between nanostructure and Aβ provided possibility for nucleophilic substitution reaction between nanostructure and Lys^16^.

Next, noncovalent interactions were measured by surface plasmon resonance (SPR) analysis. Figure 2E showed both Ni NPs and PNi NPs could bind with Aβ. Generally speaking, the lower the binding affinity constants (Kd) were, the higher Aβ‐binding affinity was. Therefore, PNi NPs with lower Kd exhibited higher Aβ‐binding affinity than Ni NPs, which might be due to that the PAm component in PNi NPs possessed π–π stacking interaction with Aβ. According to the above experiments, it was obvious that both components (NiPAm, tBAm, PAm, AAc etc.) and B6 modification in these nanoparticles were significant in the interactions between Aβ and nanoparticle. In this sense, the Aβ fibrillization inhibition effects of nanoparticles were understood. The tailor‐designed B6‐PNi NPs could bind Aβ via multivalent interactions (e.g. covalent linkage, hydrophobic interaction, π–π stacking), and the Aβ‐binding affinity of which was higher than those of B6‐Ni NPs (covalent linkage, hydrophobic interaction), PNi NPs (hydrophobic interaction, π–π stacking) and Ni NPs (hydrophobic interaction). Collectively, in virtue of Aβ_16‐20_ matching strategy induced multivalent interactions containing strong covalent linkage, B6‐PNi NPs improved Aβ‐binding affinity to reduce Aβ‐Aβ interactions for dissolution and reassembly of Aβ fibrils. Based on the aforementioned result, the rational chemical design of B6‐PNi NPs was considered as an innovative design for nanosystem in terms of inhibiting Aβ fibrillation.

### 2.4 Reduction of Aβ‐mediated neurotoxicity by B6‐PNi NPs

**FIGURE 3:**
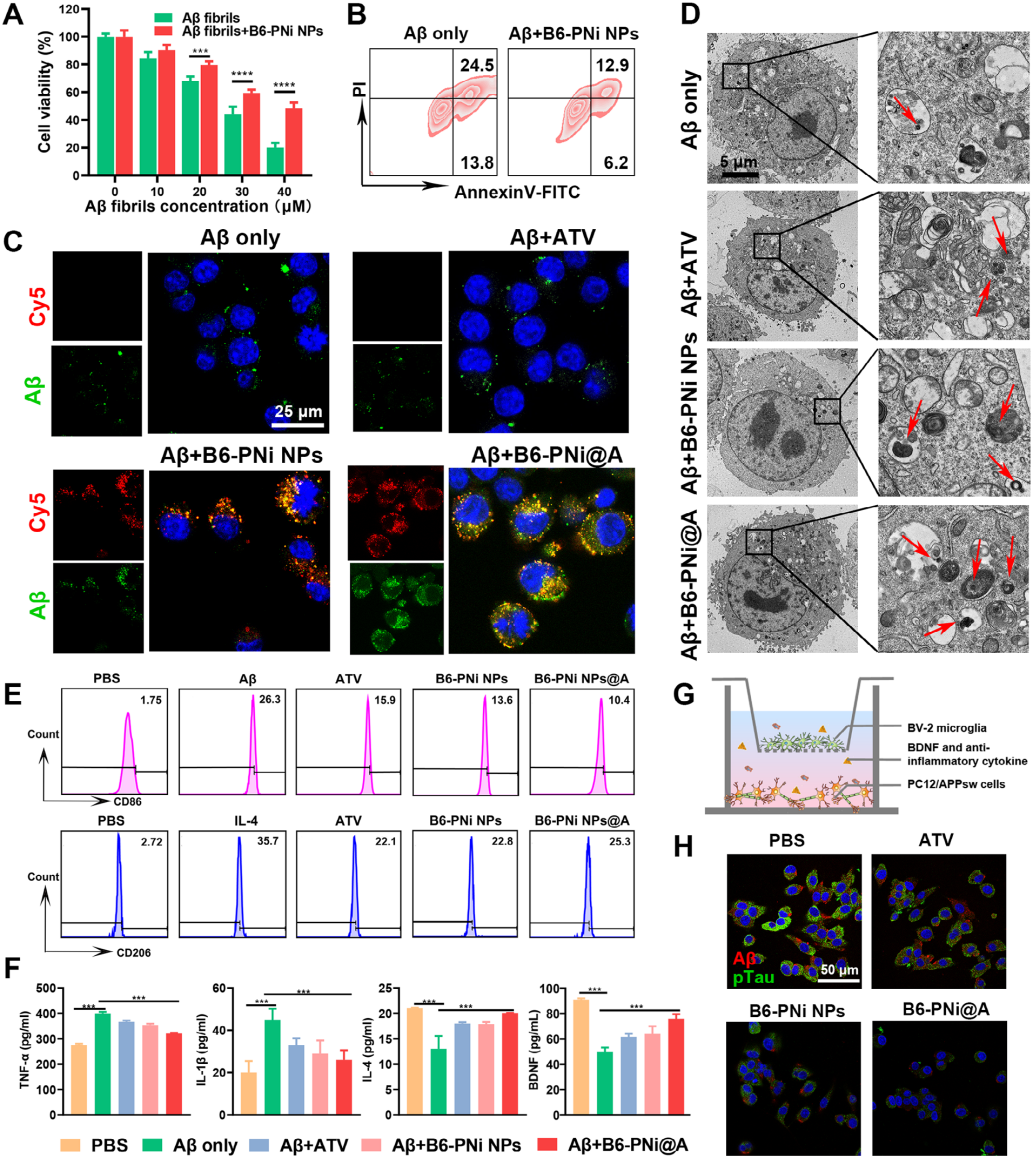
Effect of B6‐PNi@A on Aβ induced neurotoxicity and microglial dysfunction. (A) Aβ fibrils mediated neuron‐like PC12 neurotoxicity (*n* = 5). (B) Flow cytometric assay of apoptosis in PC12 cells. Lower right quadrant and upper right quadrant represented early and late apoptosis stages, respectively. (C) Confocal laser scanning microscope images showed the phagocytosis of Aβ and B6‐PNi NPs in microglia. Aβ and B6‐PNi NPs were labeled with FITC (green) and Cy5 (red), respectively. (D) Representative bio‐TEM images to show Aβ phagocytosis in BV‐2 microglia. Red arrows indicated Aβ accumulations in lysosomes. (E) Flow cytometric results showing CD86 and CD206 expression on BV‐2 cells. (F) ELISA data of TNF‐α, IL‐1β, IL‐4, and BDNF secreted from microglia (*n* = 3). (G) The design scheme of the transwell system based neuron‐microglia co‐culture model. (H) Fluorescence images to show intracellular Aβ (red) and hyperphosphorylated tau (pTau, green) expressions in PC12/APPsw cells. *p < 0.05, **p < 0.01, ***p < 0.001.

Previous studies reported that toxic Aβ fibrils tended to attack neurons for cell death.^[^ ^17^ ^]^ Then PNi NPs with Aβ fibrillization inhibition effect were used to explore the effect on neurons. Firstly, Aβ monomers were incubated alone or with B6‐PNi NPs in medium containing H_2_O_2_ to form Aβ fibrils or Aβ&PNi NPs, respectively. When neuron‐like rat pheochromocytoma (PC12) cells were exposed to the above Aβ aggregations, cell viability was detected. As illustrated in Figure 3A, Aβ fibrils induced neurotoxicity in concentration‐dependent manner. In contrast, the B6‐PNi NPs treatment improved neuronal survival with higher cell viability. What's more, the apoptosis level was also evaluated (Figure 3B). After B6‐PNi NPs treatment, the apoptotic cells reduced from 38.3% to 19.1%. These results substantiated that B6‐PNi NPs relieved Aβ‐mediated neurotoxicity and possessed neuroprotection effect.

### 2.5 B6‐PNi@A normalized microglial immunologic dysfunction for Aβ clearance

Microglia as intrinsic cerebral immune cell is responsible for Aβ clearance.^[^ ^18^ ^]^ Unfortunately, microglia is activated by toxic Aβ fibrils to a proinflammatory state, which exhibits Aβ phagocytosis disorder as well as promoted neuronal Aβ secretion.^[^ ^10^ ^]^ Herein, atorvastatin (ATV) as immunomodulator was loaded into B6‐PNi NPs in this work with high loading efficiency (14.4%). Western blotting assay in Figure S8 showed that Aβ group down‐regulated the expression of Aβ phagocytic receptor (scavenger receptor class A, SRA) in murine microglial cells (BV‐2). In contrast, other groups restored levels of SRA in different degrees. Especially, the SRA level in B6‐PNi@A group was 3.4‐fold higher than Aβ group, normalizing microglial phagocytosis dysfunction. And the Aβ phagocytosis of BV‐2 was further tested by confocal laser scanning microscope (Figure 3C). For FITC‐Aβ group, Aβ fibrils mainly adhered to the surface of cell membrane but not in cytoplasm, which could be explained to that Aβ fibrils down‐regulated SRA to hinder Aβ phagocytosis.^[^ ^19^ ^]^ However, ATV treatment promoted FITC‐Aβ uptake into cytoplasm, these results confirmed that ATV with anti‐inflammatory property recovered SRA expression by normalizing microglial phagocytosis dysfunction. As for B6‐PNi NPs group, green FITC labeled Aβ and red Cy5 labeled B6‐PNi NPs fluorescence signals were co‐localized inside cells. It was reasonable that the B6‐PNi NPs prevented Aβ fibrillation initially and the reassembled Aβ&PNi NPs were spontaneously internalized into cells. Not surprisingly, B6‐PNi@A group showed the maximum fluorescence intensity of colocalization. These results suggested that B6‐PNi@A restored Aβ phagocytosis ability of microglia for Aβ clearance through Aβ fibrillization inhibition and microglial normalization. What is more, the size (40–80 nm) of reassembled Aβ&PNi@A composites was good for microglial drug uptake via SRA mediated endocytosis, which was also an important reason for Aβ phagocytosis/clearance. Subsequently, bio‐TEM was conducted to validate the fluorescence image results. As shown in Figure 3D, more Aβ accumulations were observed in lysosomes in B6‐PNi@A group. The above results confirmed that B6‐PNi@A promoted Aβ phagocytosis/clearance of microglia.

Next, the effect of B6‐PNi@A on the immunomodulation of microglia was investigated (Figure 3E,F). Both flow cytometric assay and enzyme linked immunosorbent assay (ELISA) results showed B6‐PNi@A reduced expressions of pro‐inflammatory markers (CD86, TNF‐α, IL‐1β) and increased expressions of anti‐inflammatory markers (CD206, IL‐4). These results illustrated that B6‐PNi@A alleviated microglia activation and normalized immunologic function, which could be due to B6‐PNi NPs mediated Aβ fibrillation inhibition and ATV mediated anti‐inflammatory property. Furthermore, the brain derived neurotrophic factor (BDNF) secretion was also increased after B6‐PNi@A treatment (Figure 3F). Considering the neuroprotection function of BDNF, we next measured the effect of B6‐PNi@A on microglia‐to‐neuron crosstalk. Transwell system based neuron‐microglia co‐culture model was established (Figure 3G). BV‐2 cells pre‐treated with Aβ fibrils or different Aβ&nanostructure composites were plated in upper compartment. PC12/APPsw cells, the PC12 cells which transfected with Aβ precursor protein (APP) bearing Swedish double mutation, were plated in lower compartment. Results (Figure 3H, Figure S9) showed that ATV, B6‐PNi NPs or B6‐PNi@A treated microglia suppressed neuronal Aβ production. Especially, western blotting results showed that B6‐PNi@A group reduced the level of Aβ to 48% in comparison with PBS group (Figure S10). These results were attributed to microglia‐to‐neuron crosstalk such as the delivery of anti‐inflammatory cytokines and BDNF.^[^ ^20^ ^]^ Microtubules involve a variety of related proteins such as tau protein and microtubule‐associated protein‐2 (MAP‐2), which play important roles in neuron movement. Since Aβ could activate PI3K/AKT/GSK‐3β signaling pathway to promote tau protein phosphorylation for microtubules damage in neuron,^[^ ^21^ ^]^ the B6‐PNi@A reduced tau phosphorylation to 54% compared to PBS group (Figure S10). In addition, the increased MAP‐2 level in Figure S11 also implied that B6‐PNi@A improved stability of microtubule structure. In summary, the above results confirmed that B6‐PNi@A normalized immunologic and neuroprotection function of microglia, further reprogramming a friendly brain environment for Aβ clearance from the source to the destination via both enhancing Aβ phagocytosis of microglia and reducing Aβ secretion of neurons.

### 2.6 BBB permeability of B6‐PNi NPs

**FIGURE 4:**
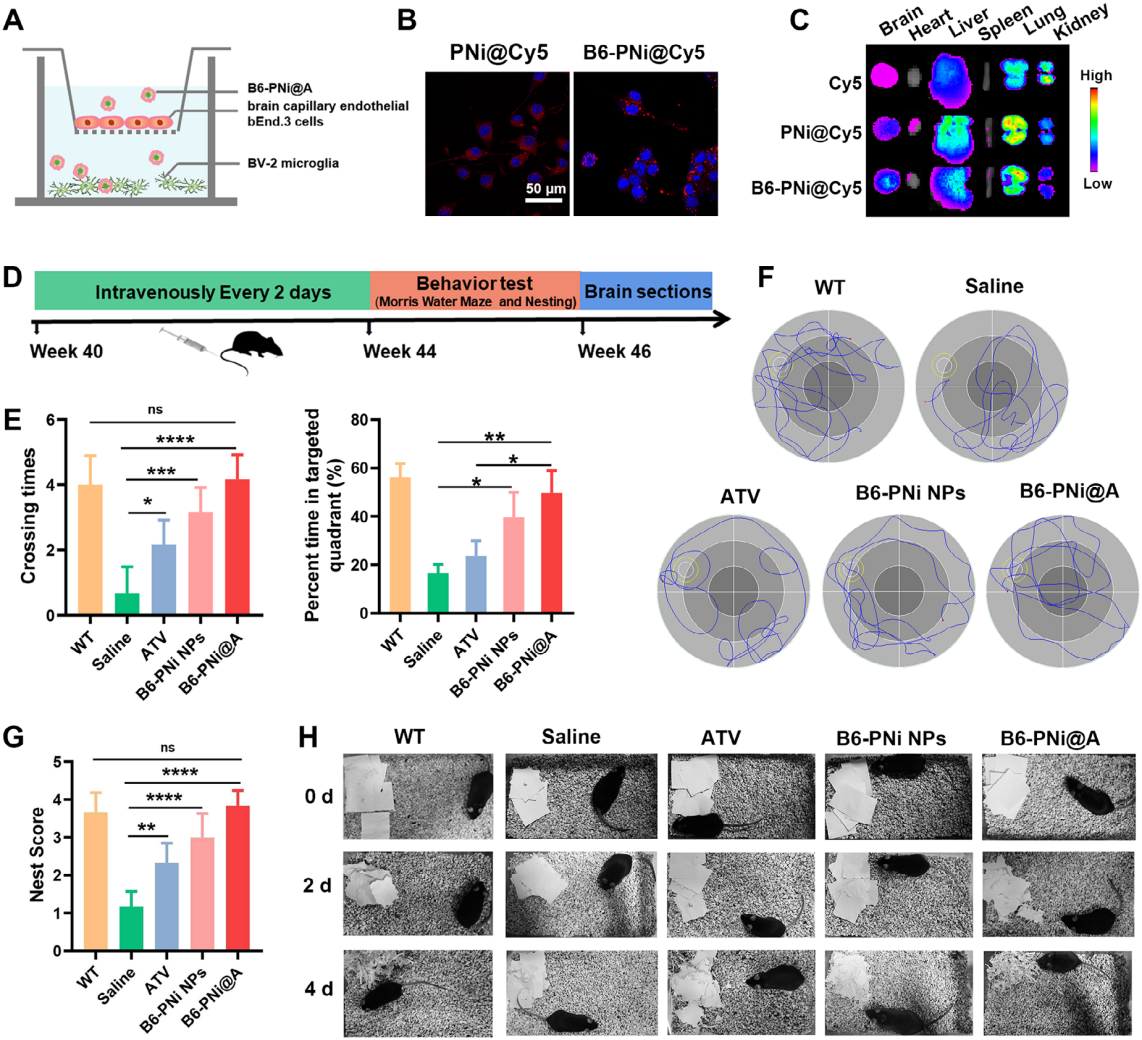
BBB permeability and behavioral evaluation of B6‐PNi@A in APP/PS1 mice. (A) The design scheme of in vitro BBB model. (B) Representative fluorescence images of BV‐2 cells in the in vitro BBB model. (C) Ex vivo fluorescence images of major tissues. (D) Experimental timeline of treatment, behavior test and brain sections. (E) Spatial memory indexes during Morris water maze experiment (*n* = 6). (F) Representative swimming paths in Morris water maze. The circle represented escape platform. (G) The nests construction experiment were scored blindly. (H) Digital images at day 0, 2 and 4 in nest construction experiment (*n* = 6). *p < 0.05, **p < 0.01, ***p < 0.001, ****p < 0.0001.

Transwell chambers seeded with a monolayer of mouse brain capillary endothelial bEnd.3 cells were established firstly to simulate in vitro BBB model (Figure 4A). PNi@Cy5 or B6‐PNi@Cy5 was incubated with bEnd.3 cells in upper compartment for 6 h, and the fluorescence of BV‐2 cells in lower compartment was measured. As shown in Figure 4B, after B6‐PNi@Cy5 treatment, the Cy5 signal in BV‐2 cells was higher than that of PNi@Cy5 group. These results indicated that B6 peptide as brain targeting moiety enhanced the permeability of nanoparticles across BBB via transferrin receptor (TfR) mediated transcytosis.^[^ ^9^ ^]^ What is more, the fluorescence images of major tissues in mice further proved the above results (Figure 4C). The brain accumulation of B6‐PNi@Cy5 was up to 1.6‐fold higher than that of PNi@Cy5. Hence, the brain targeting effect of B6‐PNi@Cy5 was expected to enhance AD therapeutic efficacy.

### 2.7 B6‐PNi NPs improved cognitive impairments, cleared Aβ, modulated microenvironment and protected neurons in APP/PS1 transgenic mice

Encouraged by the above desirable results in vitro, we wondered the feasibility in AD mice. APP/PS1 double transgenic mouse is a commonly used multitransgenic animal model that expresses two familial AD mutant genes for APP together with mutant presenilin 1 (PS1), displaying memory deficits and cognitive dysfunction. Male APP/PS1 mice were given ATV, B6‐PNi NPs and B6‐PNi@A via caudal vein injection every two days (Figure 4D). Saline treated APP/PS1 mice and wild‐type (WT) mice were included to ascertain AD‐relevant deficits in APP/PS1 mice at baseline. After treatments, the spatial memory function of mice was tested by using Morris water maze (MWM) (Figure 4E,F). After training, saline treated AD mice exhibited typical swimming path and poorly‐focused escape platform searching performance. In contrast, AD mice treated with ATV or B6‐PNi NPs showed a slighter improvement in searching strategy. Particularly, B6‐PNi@A treated mice increased number of crossing escape platform and percent time in targeted quadrant in comparison with other group, which showed similar spatial learning and memory function with WT controls. The cognitive ability of mice was measured by nest construction experiment (Figure 4G,H). The drug treated mice displayed an improved nesting ability compared with AD control mice on the second and forth days. These behavior results corroborated that B6‐PNi@A rescued cognitive and memory impairments in mice.

**FIGURE 5:**
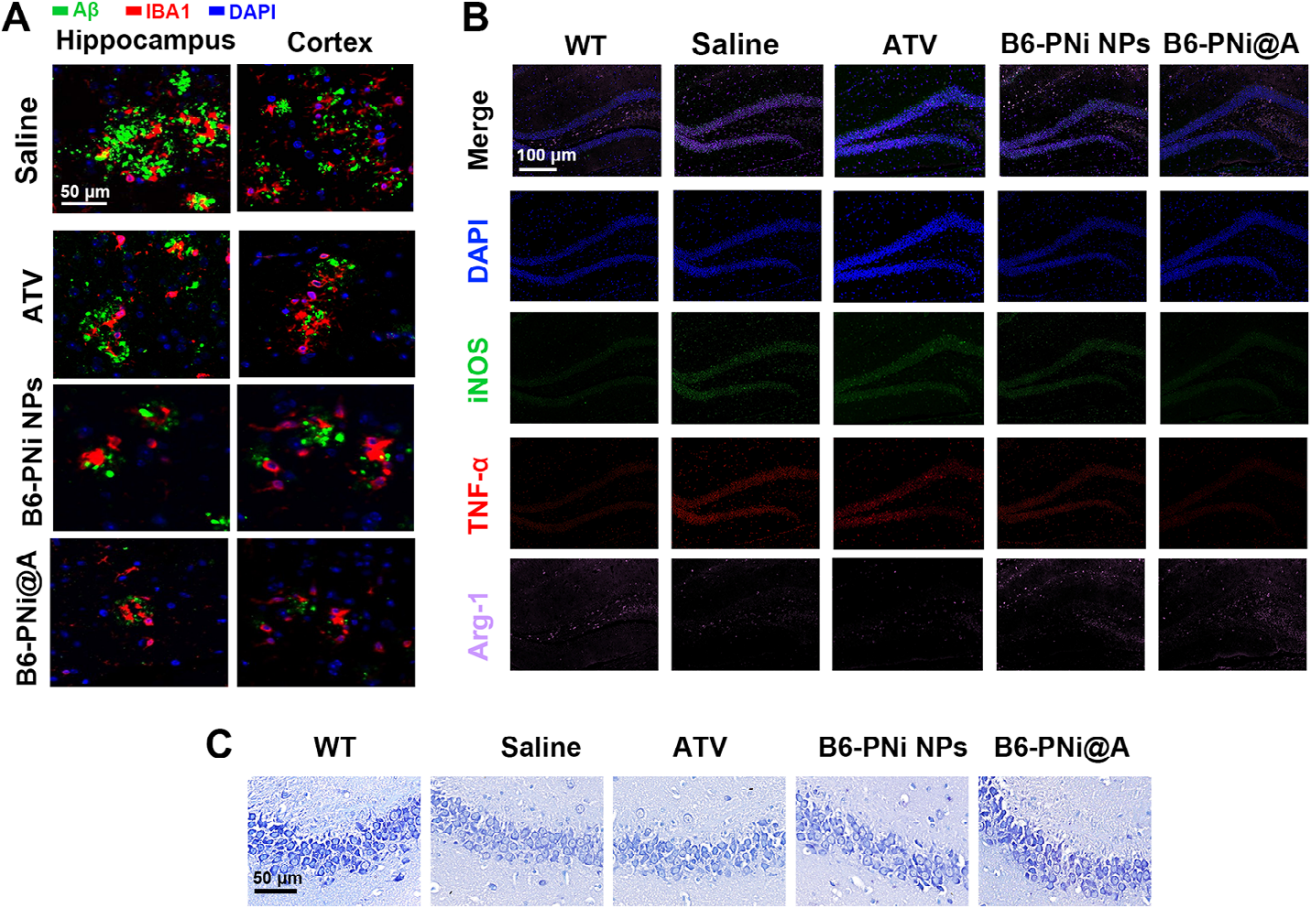
Pathologies evaluation in brain of APP/PS1 mice after different treatments. (A) Immunostaining for Aβ burden in hippocampus (left) and cortex (right), respectively. IBA1 for microglia (red), 6E10 for Aβ plaque (green). (B) Representative fluorescence images in hippocampus assessing immunologic function of microglia (blue:DAPI, green:iNOS, red: TNF‐α, purple: Arg‐1). (C) Representative data for Nissl staining of neuron in hippocampus of mice.

Next, the mechanisms underlying the AD therapeutic effect of B6‐PNi@A was investigated. Hippocampus with memory and learning function was the main lesion area with the most Aβ accumulation, which was constantly studied in AD. The immunofluorescence staining in Figure 5A showed a large number of amyloid plaques deposited in cortex and especially hippocampus of APP/PS1 mice. However, ATV, B6‐PNi NPs or B6‐PNi@A treatments decreased Aβ expression significantly. The Aβ clearance effect of B6‐PNi@A was consistent with in vitro results, which might be explained by the enhancement of microglial Aβ phagocytosis as well as reduction of neuronal Aβ production. In addition, the typical marker of microglia, IBA1, was observed adjacent to Aβ deposition (Figure 5A). Then the immunologic function of microglia was further assessed. Figure 5B showed that B6‐PNi@A increased arginase‐1 (Arg‐1), reduced iNOS and TNF‐α, which implied the anti‐inflammatory microglia polarization and further AD microenvironment normalization. Moreover, hyperphosphorylated tau expression was also evaluated, and results showed B6‐PNi@A inhibited tau phosphorylation obviously (Figure S12).

Necrotic cell death was evaluated by Nissl staining (Figure 5C). For WT group, there were clearly visible nucleolus and a large amount of Nissl bodies in the cyto‐plasm. However, APP/PS1 mice decreased Nissl bodies and caused cell swelling. In contrast, B6‐PNi@A treatment increased number of Nissl bodies, alleviated the impairment of neuronal loss and shrinkage. In addition, Nissl staining was scored by using a semi‐quantitative grading system. It showed that B6‐PNi@A significantly reduced the Nissl score in Figure S13. These results revealed the Aβ clearance would protect neurons significantly. What is more, the in vivo biocompatibility of B6‐PNi@A was measured. H&E staining in major tissues showed no obvious difference in pathology among groups (Figure S14). And no abnormalities in body weight and food uptake were observed. These results indicated the potential biosafety for clinical applications.

## 3 CONCLUSION

In this study, we have tailor‐designed a dissociable nanosystem based on Aβ sequence‐matching strategy. B6‐PNi@A (80–100 nm) with rational chemical design could decompose into PNi NPs (10 nm) in AD microenvironment. Meanwhile, PNi NPs match Aβ with high binding affinity via multivalent interactions, including covalent interaction, hydrophobic interaction and π‐π stacking. Notably, the controllable covalent interaction between nanostructure and Aβ is an innovative design for Aβ fibrillation inhibition. The high Aβ‐binding affinity allows the reduction of Aβ–Aβ interactions to disassemble Aβ fibrils into Aβ monomers. What is more, Aβ monomers and nanostructures reassemble into Aβ&nanostructure composite, contributing to microglia targeted drug delivery for Aβ clearance. Results show that B6‐PNi@A reduces Aβ‐associated neuropathology, remodulates AD microenvironment, protects neurons and improves cognitive impairments in APP/PS1 transgenic mice. As a proof of concept, the Aβ sequence‐matching strategy is useful for nanostructure design for high Aβ‐binding affinity.

## CONFLICT OF INTEREST STATEMENT

The authors declare no conflicts of interest.

## ETHICS STATEMENT

All procedures associated with animals were performed according to the National Institutes of Health Guide for the Care and Use of Laboratory Animals while the ethics were approved by the Institutional Animal Care and Use Committee of Zhengzhou University (SCXK2021‐0009).

## Supporting information

Supporting Information

## Data Availability

All data related to this study are present in the article and in the Supporting Information. Any other data associated with this work are available from the corresponding authors upon request.
