# Supplementary material for: The dissolution, reassembly and further clearance of amyloid‐β fibrils by tailor‐designed dissociable nanosystem for Alzheimer's disease therapy
Source: Exploration (Beijing). 2023 Nov 23;4(3):20230048. doi: 10.1002/EXP.20230048 (PMC11189570; doi:10.1002/EXP.20230048)
Supplement: Supplementary file 1 — Supporting Information [file EXP2-4-20230048-s001.doc]

Supporting Information

**The dissolution, reassembly and further clearance of amyloid-β fibrils by tailor-designed dissociable nanosystem for Alzheimer’s disease therapy**

**Qianhua Feng,^1,2^ Xueli Zhang,^1^ Nan Zhang,^1,2^ Huan Gu,^3^ Ning Wang,^1^ Jing Chen,^1^ Xiaomin Yuan,^1^ Lei Wang*^1,2^**

^1^ School of Pharmaceutical Sciences, Zhengzhou University, Zhengzhou, China

^2^ Henan Key Laboratory of Targeting Therapy and Diagnosis for Critical Diseases, Zhengzhou, China

^3^ Department of Chemistry, Chemical and Biomedical Engineering, University of New Haven, West Haven, USA

**Correspondence**

Lei Wang, School of Pharmaceutical Sciences, Zhengzhou University, Zhengzhou 450001, P. R. China

Email: wanglei1@zzu.edu.cn

**EXPERIMENTAL SECTION**

**Materials**

N-Isopropylacrylamide (NiPAm), N-Phenylacrylamide (PAm), N-tert-Butylacrylamide (tBAm), N,N’-methylenebisacrylamide (Bis), acrylic acid (AAc), sodium dodecyl sulfate (SDS), ammonium persulfate (APS), atorvastatin (ATV), 1,1,1,3,3,3-hexafluoro-2-propanol (HFIP), N-(3-dimethylamino propyl-N’-ethylcarbodiimide) (EDC) and N-Hydroxysuccinimide (NHS) were purchased from Sigma-Aldrich. B6 peptide (CGHKAKGPRK) and Aβ_42_ were synthesized by GL Biochem Ltd.. Fluorescence dyes including Fluorescein isothiocyanate (FITC), Thioflavin T (ThT) and Cyanine5 (Cy5) were purchased from Aladdin Reagent (Shanghai, China). Other reagents were standard Sinopharm Chemical Reagent (Shanghai, China).

**Preparation and characterization of B6-PNi NPs**

PNi NPs were synthesized after a free-radical polymerization process. In a typical procedure, NiPAm (176.5 mg), AAc (22 μL), PAm (9.5 mg), tBAm (157 mg), Bis (10 mg, crosslinking agent), SDS (50 mg, surface active agent), ethanol (2 mL) and water (48 mL) were mixed and then purged by nitrogen. After that, the mixture was heated at 65 °C and added with APS (30 mg). The reaction was carried out for 3 h under stirring. The resulting solution was dialyzed to obtain PNi NPs. To introduce B6 peptide onto PNi NPs, 3-aminophenylboronic acid (APBA) was modified on PNi NPs firstly. PNi NPs (40 mg) were activated by EDC (13 mg) and NHS (9.6 mg) in N,N-dimethylformamide, and then reacted with APBA (10 mg) for 12 h, the sample was concentrated to obtain PNi NPs-APBA. Next, PNi NPs-APBA was stirred with dopamine (DA, 20 mg) for 24 h to obtain PNi NPs-APBA-DA. B6 peptide (20 mg) was activated by EDC (26 mg) and NHS (19.2 mg) in N,N-dimethylformamide, and then reacted with PNi NPs-APBA-DA for 12 h, the B6-PNi NPs were purified by dialysis. Ni NPs and B6-Ni NPs were synthesized without PAm according to the syntheses of PNi NPs and B6-PNi NPs, respectively. For ATV loading, ATV, NiPAm, AAc, PAm, tBAm, *etc* were mixed, and reacted to obtain B6-PNi@A, according to the syntheses of B6-PNi NPs. Samples were characterized by TEM (JEM-1200EX, Tokyo, Japan) and ^1^H NMR (Bruker 600MHz). To measure the size transformation behavior of B6-PNi NPs, B6-PNi NPs in dialysis bag were incubated in PBS with H_2_O_2_ (1 mM) at 37 °C under gentle shaking, and the amount of released boron was quantified by inductively coupled plasma mass spectrometry (ICP-MS) at regular time intervals. In addition, the morphology of B6-PNi NPs in H_2_O_2_ solution was observed by TEM.

**The effect of nanoparticles on Aβ fibrillation (TEM, CD, ThT assay)**

The purchased Aβ_42_ peptide was dissolved in HFIP to break down some aggregates. Next, the sample was redissolved in DMSO and diluted with PBS to obtain Aβ monomers. Aβ fibrils were prepared by incubating Aβ monomers at 37 °C for 3 d. For TEM imaging experiment, four nanoparticles (Ni NPs, PNi NPs, B6-Ni NPs, B6-PNi NPs, 200 μg mL^-1^) were incubated with Aβ monomer (50 μM) for 7 d in PBS containing H_2_O_2_ (1 mM), respectively. Besides, experiment of Aβ fibrils dissolution was also carried out by co-incubating samples with Aβ fibrils (50 μM) for 3 d. Then the morphology of samples was estimated by TEM. After treatments, the secondary structure of Aβ was detected by circular dichroism (CD) spectroscopy (JASCO-1500, Tokyo, Japan).

For ThT fluorescence assay, nanoparticles were mixed with Aβ monomers or fibrils in PBS containing H_2_O_2_ and then incubated at 37 °C. 10 μL of the incubated sample was withdrawn at predetermined time and mixed with 190 μL ThT (20 μM) in 96-well plate under dark for 15 min. Then fluorescence of ThT was recorded at 485 nm.

**^1^H NMR**

B6-PNi NPs and Aβ_16-22_ were mixed in dialysis bag and then incubated in PBS containing H_2_O_2_ for 12 h. Sample was freeze-dried, and the chemical structure of sample was confirmed in a 600 MHz ^1^H NMR spectroscopy.

**Mass spectrometry (MS) analysis**

To further verify the occurrence of nucleophilic substitution reaction between Lys^16^ in Aβ and AAc component in nanostructure, we synthesized AAc-APBA-DA according to the synthesis of PNi NPs-APBA-DA. AAc-APBA-DA and lysine were mixed in PBS containing H_2_O_2_. After reaction for 24 h, the above solution was analyzed by MS.

**Surface plasmon resonance (SPR) analysis**

SPR analysis was performed on a Biacore S200 instrument (GE Healthcare, USA). Firstly, Aβ_42_ monomer was immobilized onto CM5 chip. Then a series of Ni NPs and PNi NPs with different concentrations (15.625, 31.25, 62.5, 125, 250 μg mL^-1^) were injected into the flow system, respectively. At last, the binding affinity constants were calculated.

**Aβ-mediated neurotoxicity assay**

Aβ monomers were incubated alone or with B6-PNi NPs in medium containing H_2_O_2_ to form Aβ fibrils or Aβ&PNi NPs, respectively. Neuron-like rat pheochromocytoma (PC12) cells were seeded 5 × 10^3^ per well in 96-well plates and incubated overnight. Aβ fibrils or Aβ&PNi NPs (Aβ concentration: 0, 10, 20, 30, 40 μM, B6-PNi NPs: 16.6 μg mL^-1^) were added and incubated for 24 h, respectively. Then cell viability was measured by using MTT assay. Additionally, apoptosis level was also measured. PC12 cells were incubated with Aβ aggregations (Aβ: 20 μM, B6-PNi NPs: 16.6 μg mL^-1^) for 24 h. Then cells were stained with Annexin-V-Fluos Staining kit and the apoptosis was detected by flow cytometric analysis.

**Aβ phagocytosis of microglia**

Aβ monomers were incubated alone or with ATV, B6-PNi NPs, B6-PNi@A in medium containing H_2_O_2_ to form Aβ fibrils or Aβ&nanostructure composites, respectively. Murine microglial (BV-2) cells (1.5 × 10^5^ per well) were plated and incubated with different Aβ aggregates (Aβ: 20 μM, B6-PNi NPs: 16.6 μg mL^-1^, ATV: 5 μM) for 6 h, respectively. CLSM was used to observe the fluorescence in cells. To further measure Aβ phagocytosis in BV-2 cells, bio-TEM was applied. Briefly, after treatment with Aβ aggregates, BV-2 cells were immobilized with osmium and further dehydrated with acetone. Subsequently, cells were incubated with pure resin at 4 °C overnight. Finally, they were picked up with copper grids and stained with uranyl acetate (4%) for TEM imaging (Technei G2 20 TWIN).

**ELISA assay**

BV-2 cells (3 × 10^5^ per well) were seeded and incubated overnight. Then different Aβ aggregates were added and incubated for 24 h, respectively. Levels of TNF-α, IL-1β, IL-4 and BDNF in supernatants were examined by using ELISA technology according to the manufacturers’ instructions.

**The effect of B6-PNi@A pre-treatment of microglia on neuron**

Transwell system based neuron-microglia co-culture model was established. BV-2 cells pre-treated with Aβ fibrils or different Aβ aggregates were plated in upper compartment. PC12/APPsw cells, the PC12 cells which transfected with Aβ precursor protein (APP) bearing Swedish double mutation, were plated in lower compartment. After co-culturing for 24 h, PC12/APPsw cells were incubated with primary antibodies anti-Aβ, anti-pTau396, anti-MAP-2 and appropriate secondary antibodies. At last, fluorescence images were taken under a fluorescence microscope.

***In vitro* BBB permeability of B6-PNi NPs**

Transwell system was carried out to simulate in vitro BBB model. Mouse brain capillary endothelial bEnd.3 cells and BV-2 cells were plated in upper and lower compartments, respectively. Subsequently, Cy5 labeled PNi NPs and B6-PNi NPs were introduced into the upper compartments and incubated for 6 h. Then BV-2 cells were analyzed by fluorescence microscope.

***In vivo* distribution**

All animal experiments were approved by the Institutional Animal Care and Use Committee of Zhengzhou University. To track *in vivo* distribution of nanoplatform, Cy5 was loaded into PNi NPs and B6-PNi NPs according to the preparation method mentioned above, respectively. Mice were treated with Cy5, PNi@Cy5 and B6-PNi@Cy5 (Cy5: 100 μg kg^-1^) via caudal vein injection, respectively. The Cy5 signal was acquired by fluorescence imaging system.

**Drug treatment of APP/PS1 mice**

Male APP/PS1 transgenic mice (40 weeks old, *n*=6) were *i.v.* injected with ATV, B6-PNi NPs and B6-PNi@A (B6-PNi NPs: 21 mg kg^-1^, ATV: 3.5 mg kg^-1^) every two days for 4 weeks. Saline treated APP/PS1 mice and wild-type (WT) mice were included as control.

**Behavior analysis**

For Morris water maze (MWM), the escape platform was located in the northwest of the maze. During the training session, mice were placed in water and allowed to swim to reach the escape platform within 60 s. If mouse failed to find platform, it was guided and kept on platform for 15 s. Acquisition trials were carried out 4 times daily for consecutive 5 days. On the sixth day, mice received tests without platform and were allowed to swim for 60 s, and performance of mice was recorded. For nest construction experiment, mice were individually caged. 10 sheets of paper (5 cm by 5 cm) were introduced inside cage. We took photos on day 0, 2, 4 to compare the bite marks of each mouse. Then nesting results were scored blindly.

**Immunostaining and histology**

After MWM experiment, mice were sacrificed, brains were removed, fixed in formalin, and then embedded in paraffin. Paraffin-embedded brain tissues were incubated with primary antibodies including anti-Aβ, anti-IBA1, anti-pTau396, anti-iNOS, anti-Arg-1, anti-TNF-α overnight and further appropriate secondary antibodies. In addition, brain tissues were also taken for Nissl staining. Other tissues were removed for H&E staining and examined under microscope.

**Statistical analysis**

Results were expressed as Mean ± S.D. from at least three independent measurements. All data sets were analyzed by using GraphPad Prism6 software. One-way ANOVA and Student’s *t* test were used for multiple-comparison and two group comparisons, respectively. The level of significance was set at probabilities of **P* < 0.05, ***P* < 0.01, ****P* < 0.001 and *****P* < 0.0001.

**SUPPLEMENTARY DATA**


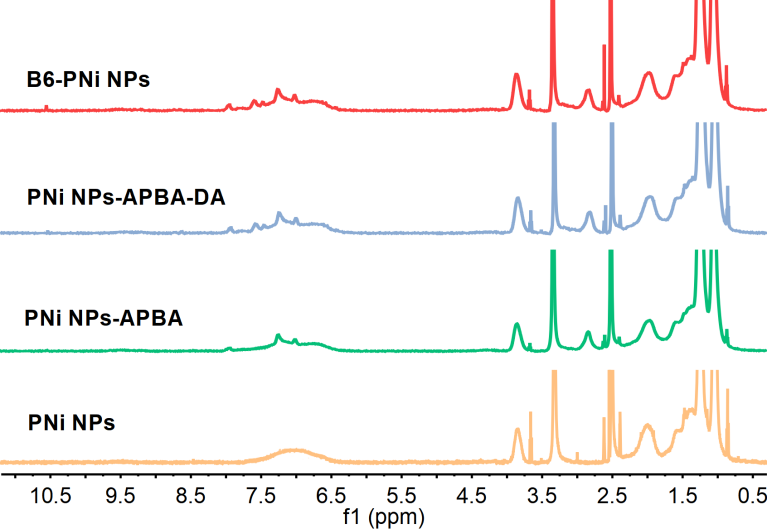


**Figure S1.** ^1^H NMR spectra of PNi NPs, PNi NPs-APBA, PNi NPs-APBA-DA, and B6-PNi NPs.


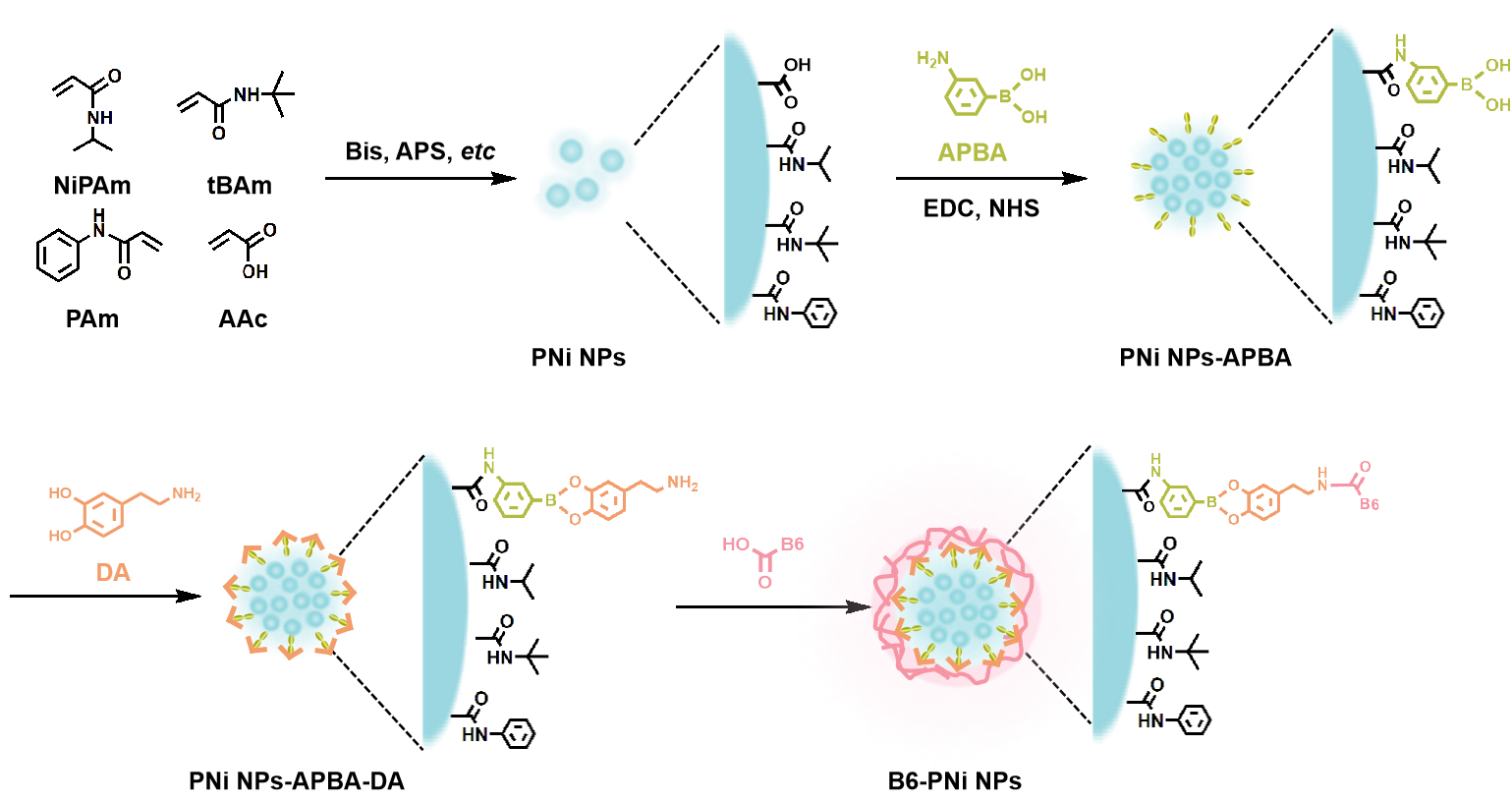


**Figure S2.** Schematic route of B6-PNi NPs.


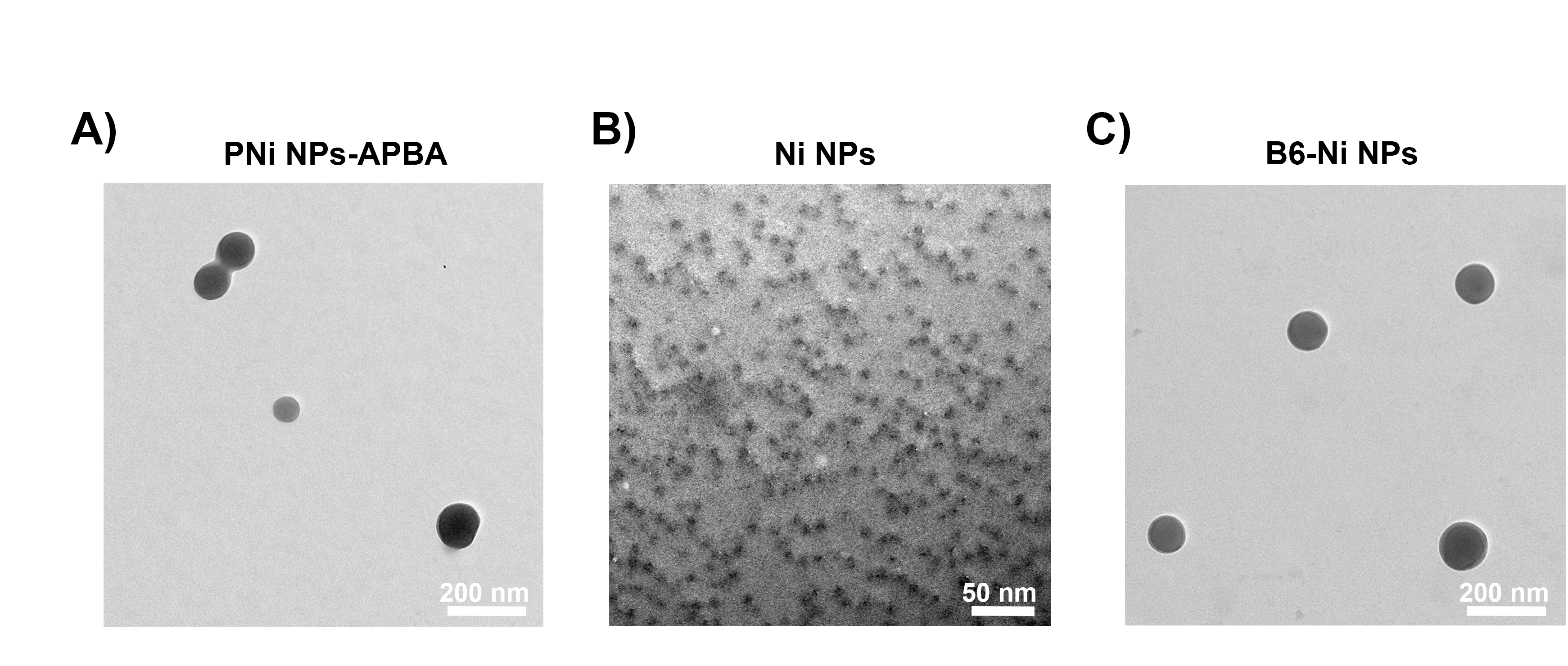


**Figure S3.** TEM image of PNi NPs-APBA.


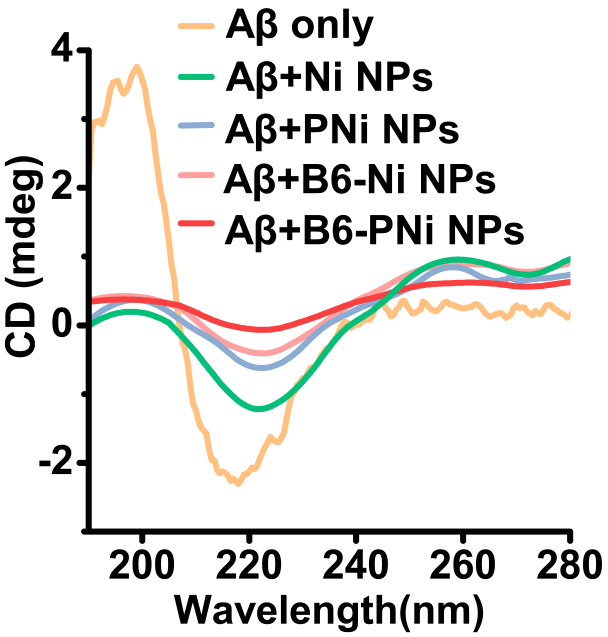


**Figure S4.** CD spectra. Different nanoparticles were incubated with Aβ mononers to inhibit Aβ fibrillation.


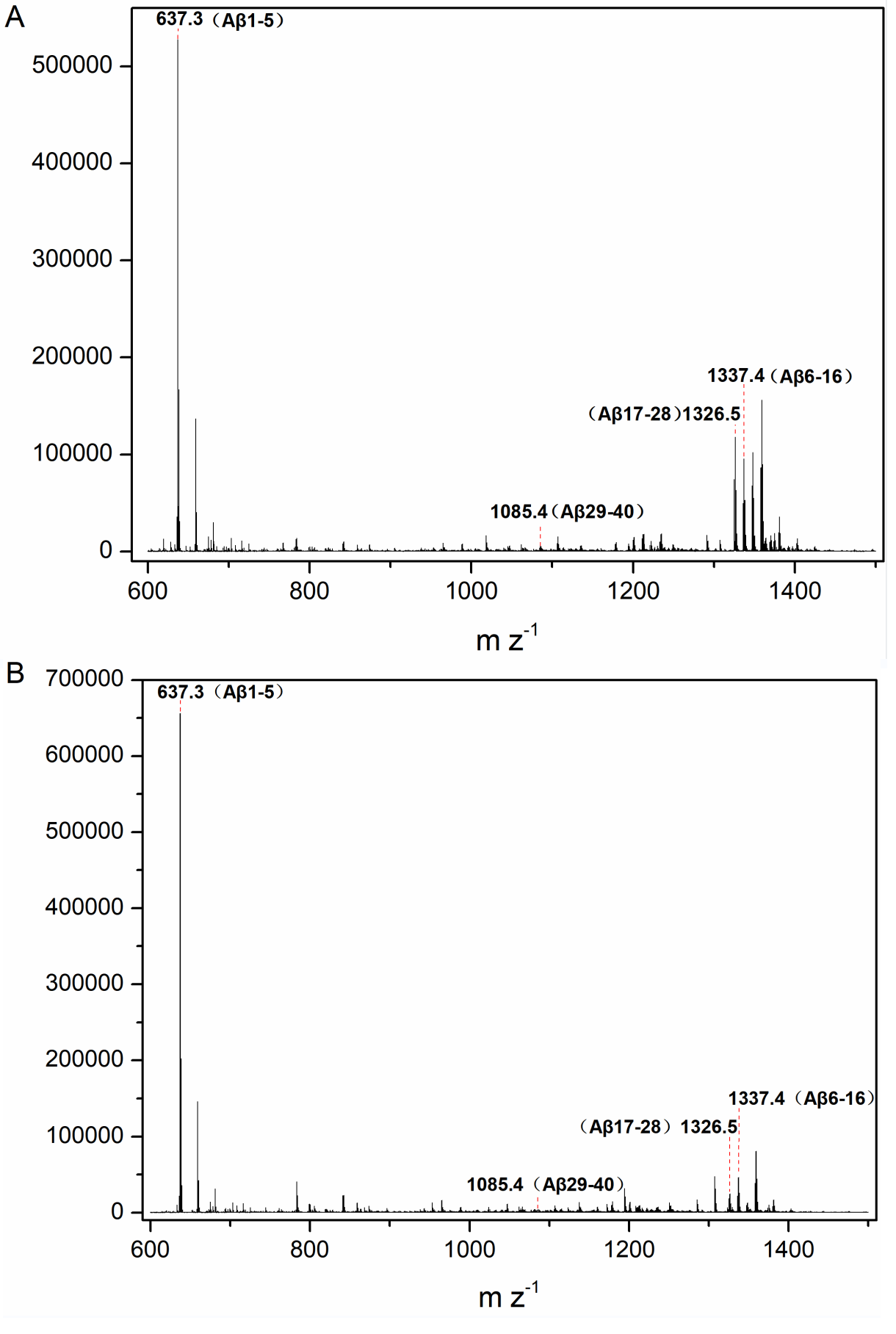


**Figure S5** Mass spectra of Aβ in absence (A) or presence (B) of B6-PNi NPs after trypsin treatment.


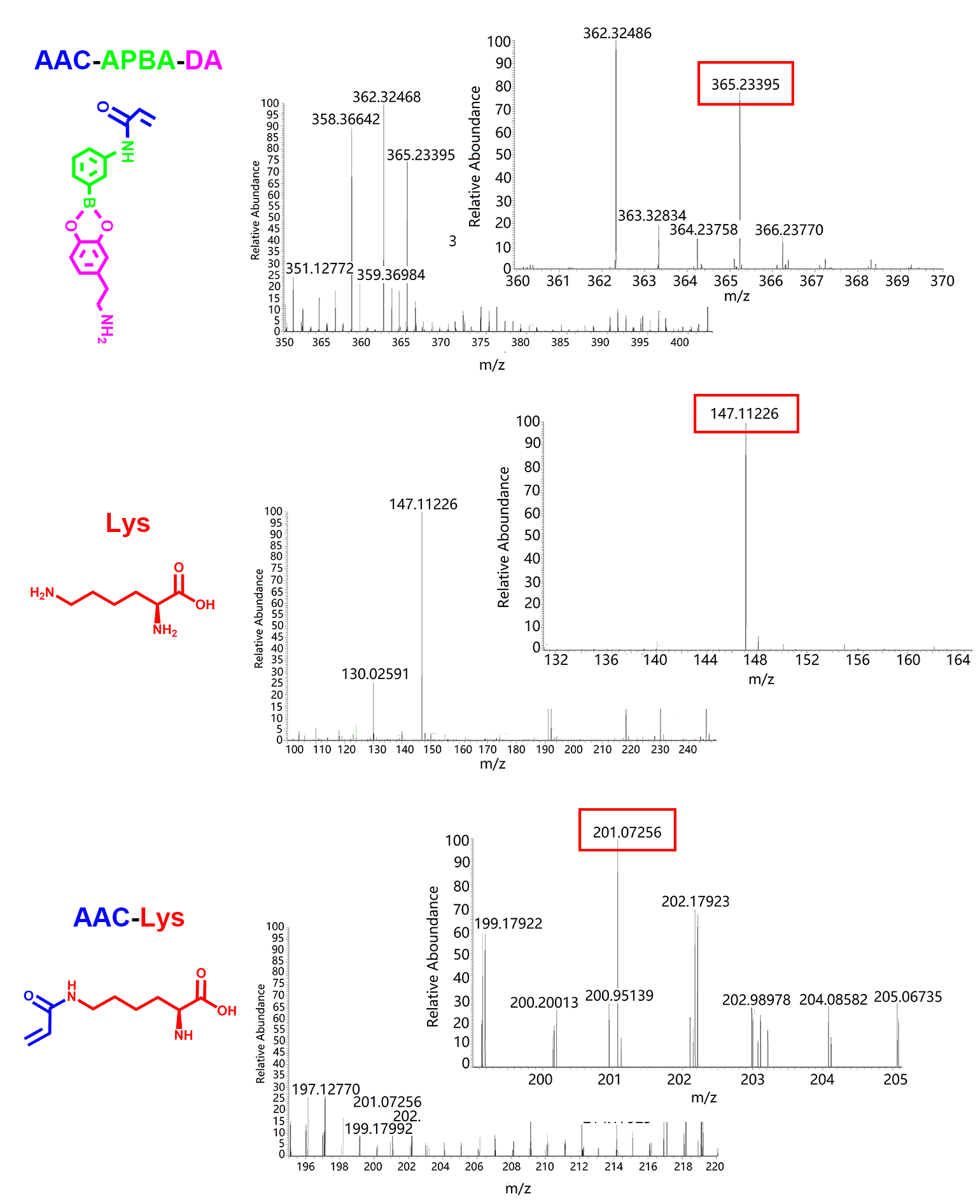


**Figure S6.** Mass spectra of AAc-APBA-DA (top), Lys (medium), and the product (AAc-Lys, bottom) after reaction between AAc-APBA-DA and Lys.


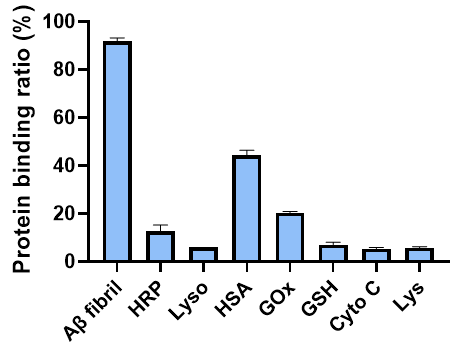


**Figure S7.** Protein binding ratio between B6-PNi NPs and different proteins containing horseradish peroxidase (HRP), lysozyme (lyso), human serum albumin (HSA), glucose oxidase (GOx), glutathione (GSH), Cytochrome C (Cyto C), lysine (Lys).


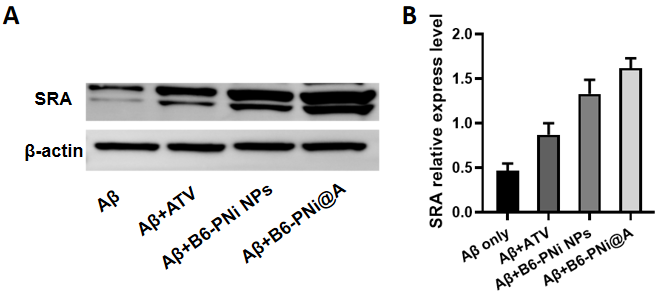


**Figure S8.** Western blotting assay to assess the levels of SRA in BV-2 microglia. The corresponding quantified results are displayed.


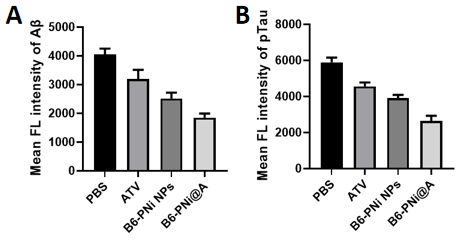


**Figure S9.** Mean fluorescence intensity of Aβ and pTau in PC12/APPsw cells.


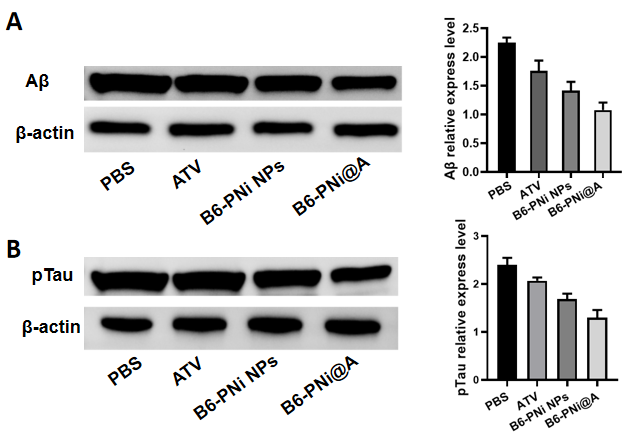


**Figure S10.** Western blotting assay to assess level of Aβ (A) and pTau (B) in PC12/APPsw cells.


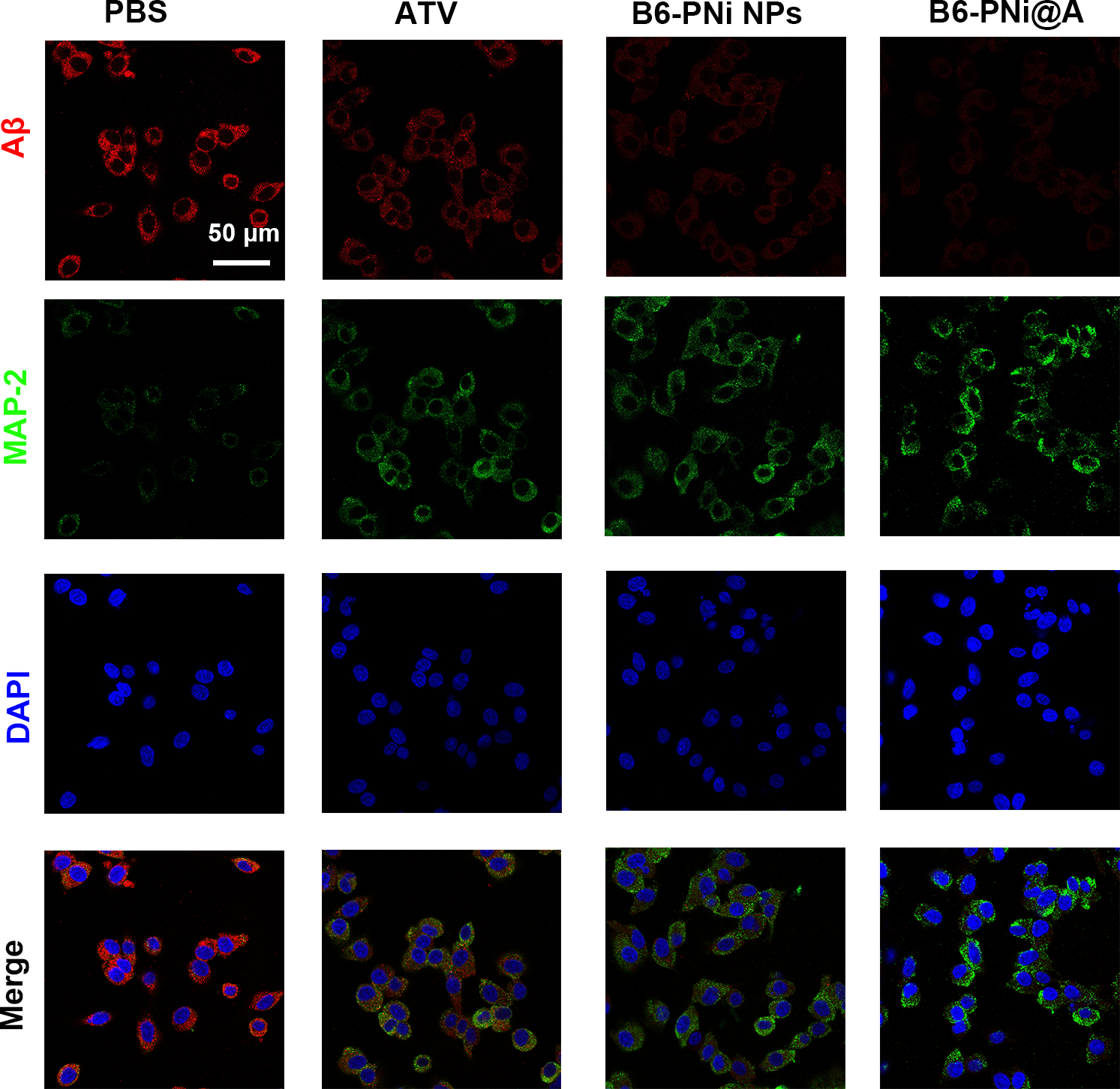


**Figure S11.** Confocal laser scanning microscope was used to measure intracellular Aβ and MAP-2 in PC12/APPsw cells. Aβ: red; MAP-2: green; DAPI: blue.


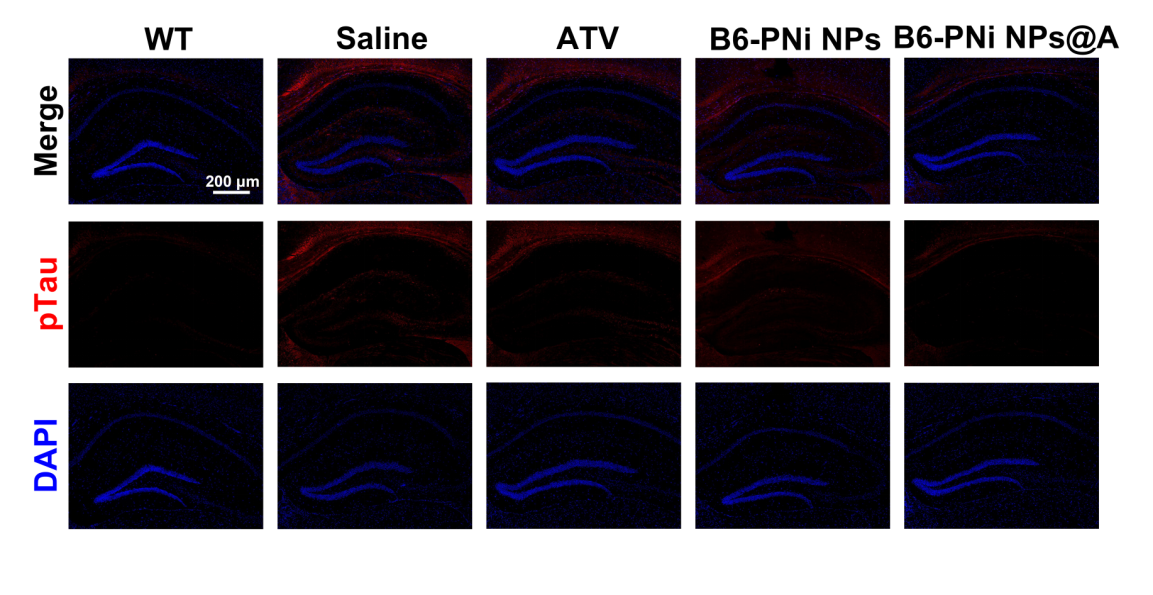


**Figure S12.** Representative phosphorylated tau (pTau) immunostaining micrographs in hippocampus.


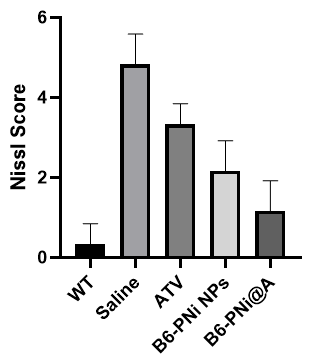


**Figure S13.** Nissl score.


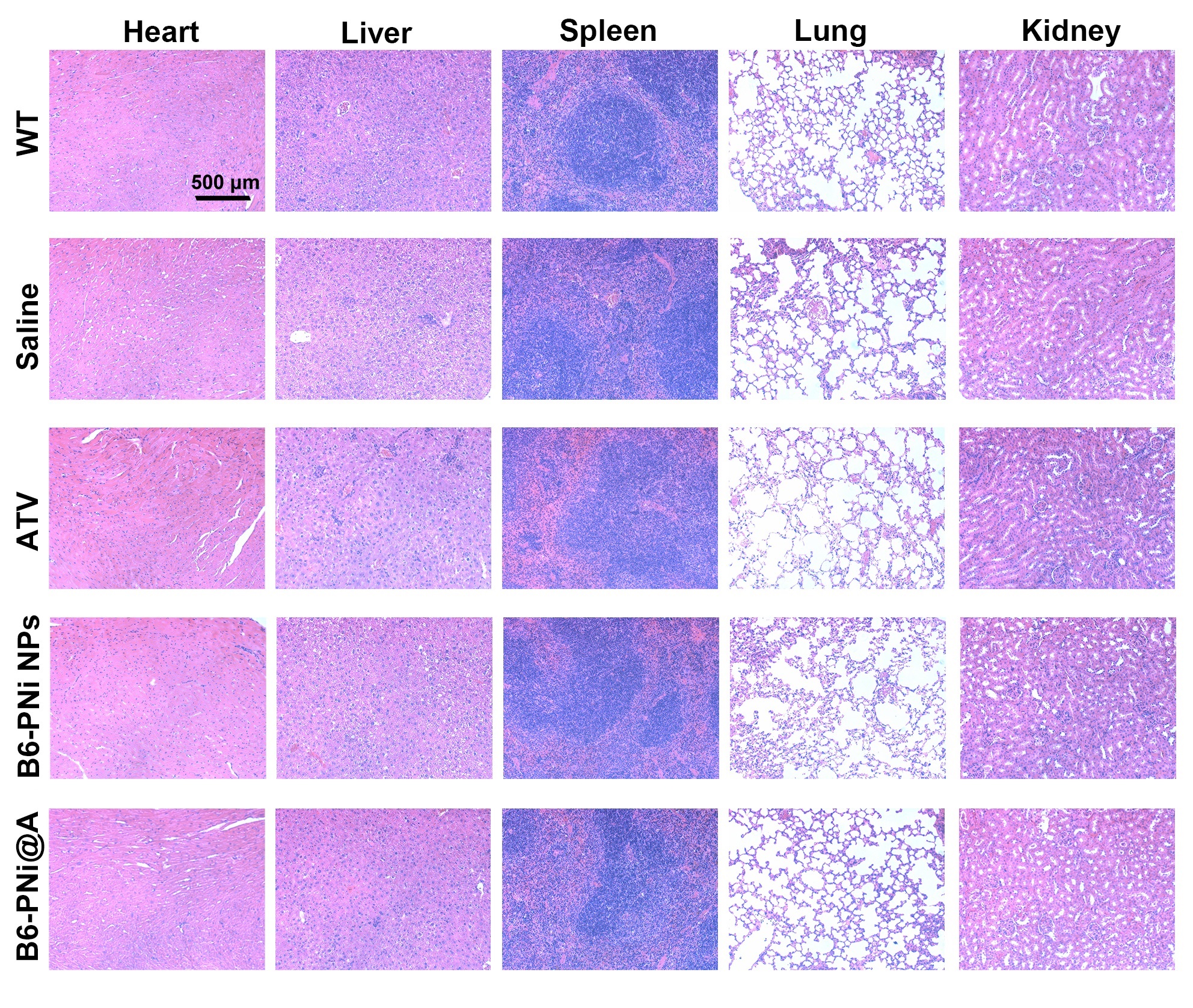


**Figure S14.** H&E staining images of tissues (heart, liver, spleen, lung, kidney) after treatments.
